# Supplementary material for: Unexpected Slow Kinetics of Poly(Methacrylic Acid) Phase Separation in the Semi-Dilute Regime
Source: Polymers (Basel). 2022 Nov 3;14(21):4708. doi: 10.3390/polym14214708 (PMC9654023; doi:10.3390/polym14214708)
Supplement: Supplementary file 1 [file polymers-14-04708-s001.zip › polymers-1968604-supplementary.pdf]

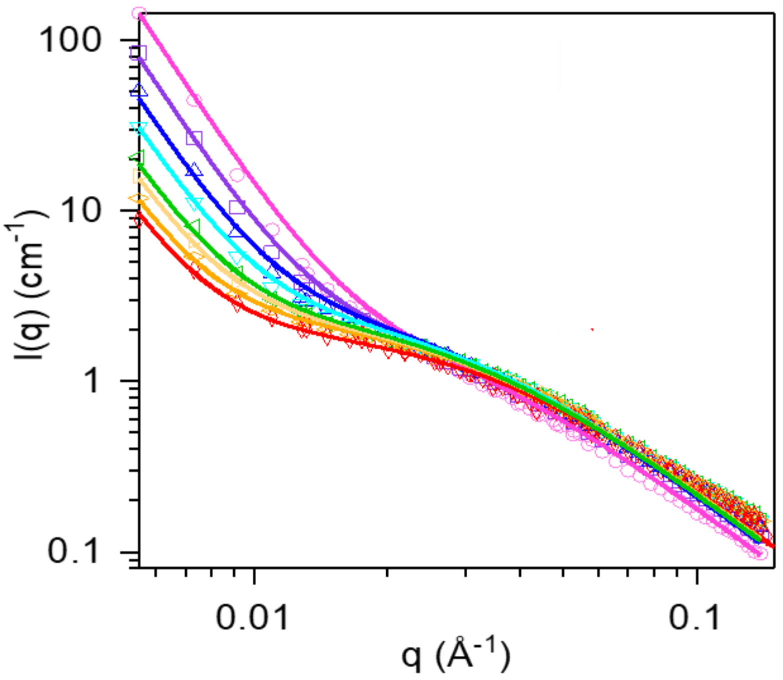

Figure S1: *SANS intensity I(q) as a function of q for a solution of PMAA in water at a volume fraction of 0.065. The time evolution of I(q) has been monitored following a temperature jump from 25°C to T_{CP} +10°C. The various delay between the temperature change and the acquisition of I(q) are represented in different colors:* ○ *60 min,* □ *180 min,* Δ *300 min,* ∇ *420 min,* ⊲ *540 min,* ⊳ 660 min, ◊ 780 min, ◊ 900 min

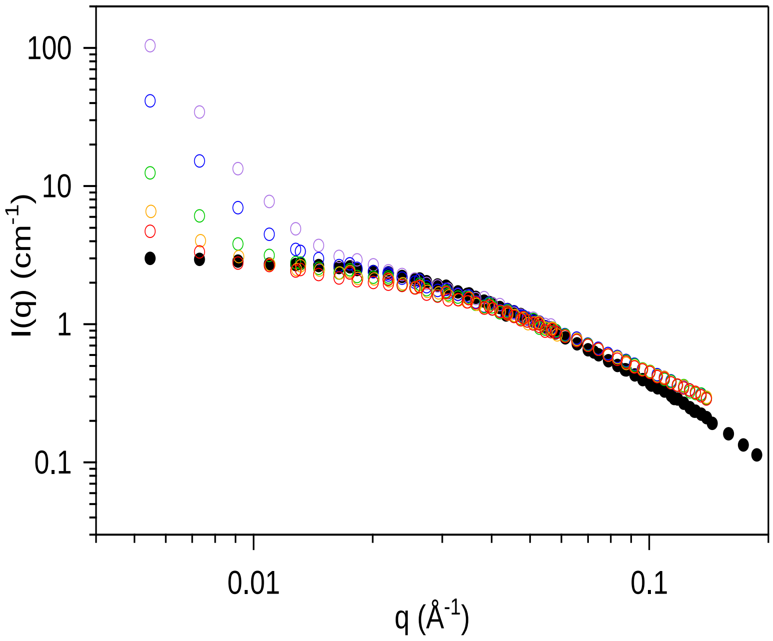

Figure S2: *SANS intensity I(q) as a function of q for a solution of PMAA in water at a volume fraction of 0.104. The time evolution of I(q) has been monitored following a temperature jump from 25°C to* T_{CP} *+10°C. The various delay between the temperature change and the acquisition of I(q) are represented in different colors:* ○ *60 min,* ○ *180 min,* ○ *300 min,* ○ *420 min,* ○ *540 min. The SANS intensity obtained for the same solution at 25°C, before the temperature change, has been included for sake of comparison (■)*

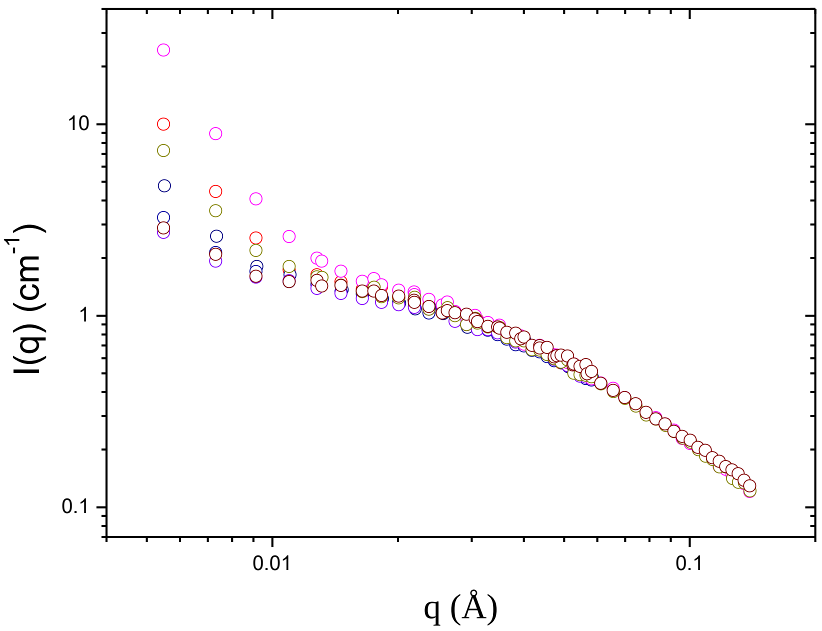

Figure S3: *SANS intensity I(q) as a function of q for a solution of PMAA in water at a volume fraction of 0.017. The time evolution of I(q) has been monitored following a temperature jump from 25°C to* T_{CP} *+10°C. The various delay between the temperature change and the acquisition of I(q) are represented in different colors:* ○ *60 min,* ○ *120 min,* ○ *180 min,* ○ *240 min,* ○ *300 min,* ○ *360 min,* ○ *420 min..*

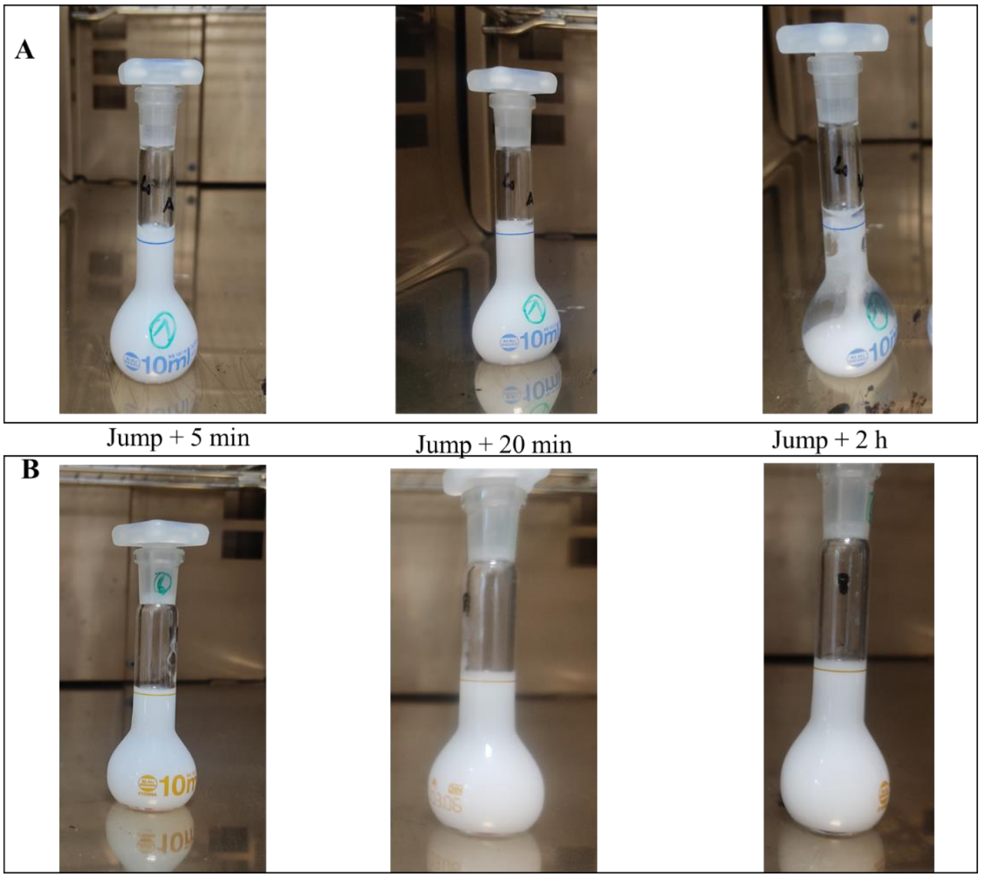

Figure S4: *Photographs of two PMAA solutions in an oven above the cloud after 3 different delays following a temperature jump (5 minutes, 20 minutes and two hours): (A)* $\phi_{PMAA} = 0.013$ *and (B) and of* $\phi_{PMAA} = 0.090$*.*
